# The GEA pipeline for characterizing *Escherichia coli* and *Salmonella* genomes

**DOI:** 10.1038/s41598-024-63832-z

**Published:** 2024-06-10

**Authors:** Aaron M. Dickey, John W. Schmidt, James L. Bono, Manita Guragain

**Affiliations:** 1 US Department of Agriculture, Agricultural Research Service, US Meat Animal Research Center, 844 Rd 313, PO Box 165, Clay Center, NE 68933 USA; 2 US Department of Agriculture, Agricultural Research Service, Eastern Regional Research Center, 600 East Mermaid Lane, Wyndmoor, PA 19038 USA

**Keywords:** Computational biology and bioinformatics, Microbiology, Molecular biology

## Abstract

*Salmonella enterica* and *Escherichia coli* are major food-borne human pathogens, and their genomes are routinely sequenced for clinical surveillance. Computational pipelines designed for analyzing pathogen genomes should both utilize the most current information from annotation databases and increase the coverage of these databases over time. We report the development of the GEA pipeline to analyze large batches of *E. coli* and *S. enterica* genomes. The GEA pipeline takes as input paired Illumina raw reads files which are then assembled followed by annotation. Alternatively, assemblies can be provided as input and directly annotated. The pipeline provides predictive genome annotations for *E. coli* and *S. enterica* with a focus on the Center for Genomic Epidemiology tools. Annotation results are provided as a tab delimited text file. The GEA pipeline is designed for large-scale *E. coli* and *S. enterica* genome assembly and characterization using the Center for Genomic Epidemiology command-line tools and high-performance computing. Large scale annotation is demonstrated by an analysis of more than 14,000 *Salmonella* genome assemblies. Testing the GEA pipeline on *E. coli* raw reads demonstrates reproducibility across multiple compute environments and computational usage is optimized on high performance computers.

## Introduction

*Salmonella enterica* (hereafter *Salmonella*) are estimated to cause at least 1 million illnesses in the United States each year^1^. *Escherichia coli* are ubiquitous in a wide variety of environments relevant to food safety including food animal gastrointestinal systems, animal production sites, human gastrointestinal systems, meats, and manure impacted soils. A small but clinically important sub-set of *E. coli* are pathogenic. The ubiquitous nature of *E. coli* contributes to their relevance beyond food safety. Due to their prominence and small genome size, *Salmonella* and *E. coli* are also two of the top organisms with available whole genome sequencing read archives (https://www.ncbi.nlm.nih.gov/sra?term=(%22public%22%5BAccess%5D)%20AND%20%22genomic%22%5BSource%5D) and assemblies (https://www.ncbi.nlm.nih.gov/genome/browse#!/overview/). Such large datasets often rely on high-performance computing to accelerate computational tasks via increased RAM, threads, and parallelization^2^. Large datasets can benefit from data analysis pipelines, which process many input files with an initial set of user specifications and distill the results to a small number of organized outputs for interpretation^3^.

Useful pipelines for epidemiologic annotation should take advantage of the most up-to-date reference information available. Actively curated reference databases meet this need by rapidly incorporating newly released genomic data. The interplay between these two dependencies can be thought of as a positive feedback loop wherein 1. Running the pipeline on new strains improves the database coverage and quality by exposing knowledge gaps and 2. The database improvement leads to more accurate search hits when running the pipeline on new strains.

Here, we introduce the GEA pipeline. GEA stands for Gammaproteobacteria Epidemiologic Annotation. Analyses central to the GEA pipeline are those using Center for Genomic Epidemiology (CGE) developed tools^4^. The databases for these tools to search are updated frequently, facilitating the positive feedback loop between our pipeline and these databases. Several existing pipelines utilize CGE developed tools^5–9^. But we are unaware of any other published pipelines, which use FimTyper^10^, MLST^11^, PlasmidFinder^12^, ResFinder^13^, SerotypeFinder^14^, and VirulenceFinder^15^ in tandem.

Another important feature of the pipeline is the use of a container. Containers allow for compute mobility^16^ and provide an increased level of reproducibility^17^. The container housing the software tools for running the GEA pipeline also takes advantage of the Scientific File System (SCIF)^18^, providing independent mount points to different apps in the container with incompatible environmental requirements.

Prior versions of the GEA pipeline have been used in published research^19–21^ in food safety, risk assessment, antimicrobial resistance gene transfer, and virulence research at the US Meat Animal Research Center. This demonstrates the utility of the pipeline and the benefit of the distilled annotation summary across hundreds of genomes. This paper describes the methods used for creating the pipeline and provides a kilo-scale demonstration of the pipeline on *S. enterica* assemblies. The pipeline is available from github.com/Phylloxera/GEA-dev.

## Results

### Computer resource usage

Table 1 provides the usage summary for testing. The run time varied from 5.04 to 34.56 h (3.15 to 21.6 min-per-*E*.-*coli*-library). Assembly accounted for 89–95% of pipeline run time.

**Table 1:** Computational resources used for GEA Pipeline testing.

Computer name	CPUs	RAM (GB)	OS	Job scheduler	HPC	Apptainer version	Run time for assembly/other steps (minutes-per-*E.*-*coli*-library)
Moose	64	1986	Oracle linux server 8.8	NA	Yes	1.2.3	2.84/0.32
Ceres	72	360	AlmaLinux 9.2 (Turquoise Kodkod)	slurm	Yes	1.2.2	2.88/0.34
Atlas	48	360	CentOS Linux 7 (Core)	slurm	Yes	1.1.6	2.86/0.29
Desktop	12	20	Virtual machine running Ubuntu 22.04.3 LTS	NA	No	1.2.4	20.42/1.18

### Output

The principal output of the GEA pipeline is the tab-delimited file, metadata.txt. The metadata can be opened in Excel. The number of rows corresponds to the number of assemblies annotated and the number of columns is dataset dependent. The annotation quality is expected to improve as the CGE database coverage improves over time. The metadata from the test dataset of 96 *E. coli* raw read libraries contained 444 columns and the metadata from the demonstration dataset of 14,310 *Salmonella* assemblies had 597 columns. Table 2 summarizes the components of the two metadata files. Tables 3 and 4 provide single annotation snapshots from VirulenceFinder^15^ *E. coli* and ResFinder^13^ *Salmonella* respectively, while Table 5 provides a snapshot of the all tools summary from *E. coli*. Table 5 suggests a relationship between FIM type and Antimicrobial Resistance Gene (ARG) content for the 96 *E*. *coli* libraries test dataset. The complete summary output file (GEA_ecoli_test_Ceres_metadata.txt) from USDA Ceres^22^ is provided in Supplementary Data S1.

**Table 2:** Summary of the sections of the GEA Pipeline test data and demonstration data tabular output.

	96 *E. coli* libraries test data	14,310 *Salmonella* demonstration data
File	GEA_ecoli_test_Ceres_metadata.txt in Supplementary Data S1	GEA_senterica_demo_Atlas_metadata.txt in Supplementary Data S1
All tools summary columns	28	14
MLST columns	28	28
Serotyping columns	12	9
Virulence columns	280	*E. coli* only
Resistance columns	54	475
PlasmidFinder columns	42	59
5 loci columns	0	12
All metadata columns	444	597

**Table 3:** A 7-column VirulenceFinder annotation from a three assembly subset of the 96 *E. coli* libraries test data (Supplementary Data S1).

Library	VirFactor20	VirID20	VirQ_T_Len20	VirContig20	VirContig20Pos	VirProtFunc20	VirAccNo20
SAM128598	katP	100	2211/2211	contig00087 len = 3725 cov = 20.3 corr = 0 origname = NODE_87_length_3725_cov_20.310952_pilon sw = shovill-spades/1.1.0 date = 20231107	936.. 3146	Plasmid-encoded catalase peroxidase	AB011549
SAM128599	nleA	99.9	1326/1326	contig00076 len = 5108 cov = 59.2 corr = 0 origname = NODE_76_length_5108_cov_59.248156_pilon sw = shovill-spades/1.1.0 date = 20231107	2717.. 4042	Non-LEE encoded effector A	AE005174
SAM128600	katP	100	2211/2211	contig00083 len = 3725 cov = 25.6 corr = 0 origname = NODE_83_length_3725_cov_25.644469_pilon sw = shovill-spades/1.1.0 date = 20231107	936.. 3146	Plasmid-encoded catalase peroxidase	AB011549

**Table 4:** A 7-column ResFinder annotation from a 4 assembly subset of the 14,310 *Salmonella* assemblies demonstration data (Supplementary Data S1).

Library	ResGene32	ResID 32	ResQ_T_Len32	ResContig32	Res contig32pos	Res Pred Pheno32	ResAccNo 32
GCA_ 032,340,135.1_PDT 001,912,849.1_genomic.fna	sul1	100	840/840	DAPZPL010000076.1 TPA_asm: Salmonella enterica subsp. enterica serovar Derby strain S414 isolate S414_swine_DK_2018 SAMEA113940708-rid19586193.guided.52, whole genome shotgun sequence	1663.. 2502	Sulfa methox azole	U12338
GCA_ 032,495,405.1_PDT 001,920,155.1_genomic.fna	sul2	100	816/816	ABNNMO010000136.1 Salmonella enterica subsp. enterica serovar 1,4,[5],12:i:- strain PNCS017511 SAMN37691013-rid19634173.denovo.139, whole genome shotgun sequence	1708.. 2523	Sulfa methox azole	HQ840942
GCA_ 032,495,425.1_PDT 001,920,154.1_genomic.fna	sul3	100	792/792	ABNNMP010000218.1 Salmonella enterica subsp. enterica serovar 1,4,[5],12:i:-strain PNCS017510 SAMN37691002-rid19634163.guided.163, whole genome shotgun sequence	2059.. 2850	Sulfa methoxazole	AJ459418
GCA_ 032,495,445.1_PDT 001,920,153.1_genomic.fna	sul3	100	792/792	ABNNMQ010000085.1 Salmonella enterica subsp. enterica serovar 1,4,[5],12:i:-strain PNCS017515 SAMN37691018-rid19634153.guided.80, whole genome shotgun sequence	18.. 809	Sulfa methoxazole	AJ459418

**Table 5:** A 3-column portion of the GEA Pipeline All Tools Summary for the 96 *E. coli* libraries test data (Supplementary Data S1) showing acquired antimicrobial resistance gene content by *fim* type.

Library	FimType	AqResGeneCount	AqResGeneList
SAM128606	FimH36	4	aph(6)-Id,aph(3ʺ)-Ib,aph(3ʺ)-Ib,aph(3ʺ)-Ib,aph(3ʺ)-Ib,sul2,tet(B)
SAM128608	FimH36	4	aph(6)-Id,aph(3ʺ)-Ib,aph(3ʺ)-Ib,aph(3ʺ)-Ib,aph(3ʺ)-Ib,sul2,tet(B)
SAM128609	FimH36	4	aph(6)-Id,aph(3ʺ)-Ib,aph(3ʺ)-Ib,aph(3ʺ)-Ib,aph(3ʺ)-Ib,sul2,tet(B)
SAM128625	FimH36	4	aph(6)-Id,aph(3ʺ)-Ib,aph(3ʺ)-Ib,aph(3ʺ)-Ib,aph(3ʺ)-Ib,sul2,tet(B)
SAM128626	FimH36	4	aph(6)-Id,aph(3ʺ)-Ib,aph(3ʺ)-Ib,aph(3ʺ)-Ib,aph(3ʺ)-Ib,sul2,tet(B)
SAM128645	FimH36	4	aph(6)-Id,aph(3ʺ)-Ib,aph(3ʺ)-Ib,aph(3ʺ)-Ib,aph(3ʺ)-Ib,sul2,tet(B)
SAM128646	FimH36	4	aph(6)-Id,aph(3ʺ)-Ib,aph(3ʺ)-Ib,aph(3ʺ)-Ib,aph(3ʺ)-Ib,sul2,tet(B)
SAM128676	FimH36	4	aph(6)-Id,aph(3ʺ)-Ib,aph(3ʺ)-Ib,aph(3ʺ)-Ib,aph(3ʺ)-Ib,sul2,tet(B)
SAM128693	FimH36	4	aph(6)-Id,aph(3ʺ)-Ib,aph(3ʺ)-Ib,aph(3ʺ)-Ib,aph(3ʺ)-Ib,sul2,tet(B)
SAM128613	FimH36	0	
SAM128614	FimH54	3	aph(6)-Id,aph(3ʺ)-Ib,aph(3ʺ)-Ib,aph(3ʺ)-Ib,aph(3ʺ)-Ib,tet(B)
SAM128631	FimH54	3	aph(6)-Id,aph(3ʺ)-Ib,aph(3ʺ)-Ib,aph(3ʺ)-Ib,aph(3ʺ)-Ib,tet(B)
SAM128629	FimH54	1	tet(A)
SAM128611	FimH552	4	aph(3ʺ)-Ib,blaCMY-2,sul2,tet(A)
SAM128642	FimH82	3	aph(3ʺ)-Ib,sul2,tet(A)
SAM128640	FimH82	1	tet(J)
SAM128598	FimH82	0	

79 libraries with FimH82 and 0 resistance genes are not shown.

### Demonstration

The pipeline ran on the 14,310 *S. enterica* assemblies in ~ 72 h (~ 18 s-per-*S*.-*enterica*-assembly) on the USDA/Mississippi State University Atlas cluster^22^. The complete summary output file is in GEA_senterica_demo_Atlas_metadata.txt in Supplementary Data S1.

## Discussion

A diverse set of bioinformatic tools has been developed for phenotypic prediction based on genomic data, especially for human pathogens. These tools often grow out of the requirements sought by a group of researchers. In the case of GEA, these included the desire to assemble and run CGE tools at the command-line on large numbers of strains with a single summary output and to have genome assemblies in a single directory ready for submission to NCBI.

Users have reported different results with the same input data, sometimes with analyses conducted many months apart. This is suspected to be caused by updates in the actively curated CGE databases. This has been confirmed in some instances. In the interest of reproducibility, the user has the option to update their local copy of the CGE databases and use the most up-to-date versions, or to leave the databases static for reproducibility across independent computational runs. Reproducibility has long been an aspiration of scientific analysis, however database dependent analyses may demonstrably benefit from non-reproducibility as database coverage increases with the passage of time.

In our testing phase, we sought to have reproducibility across multiple computational environments and this was largely achieved. The only difference in the outputs across high performance computers was due to the contig names assigned by shovill (https://github.com/tseemann/shovill). In all these cases, the length, coverage, and Pilon^23^ name were identical whereas shovill assigned contig integers differed by 1. Furthermore, shovill contig names included the date assembled, an additional possible source of discrepancy for runs taking place on different days. E.g., contig0020**5** len = 509 cov = 31.1 corr = 0 origname = NODE_348_length_509_cov_31.119617_pilon sw = shovill-spades/1.1.0 date = 2023110**7** on Ceres vs contig0020**4** len = 509 cov = 31.1 corr = 0 origname = NODE_348_length_509_cov_31.119617_pilon sw = shovill-spades/1.1.0 date = 2023110**1** on Moose (discrepancies in bold; example from row 15, column 128 of GEA_ecoli_test_Ceres_metadata.txt in Supplementary Data S1). The identical coverage and Pilon designation indicates that, in all cases, these were identical contigs, but that the final integer contig name assignment in shovill may not be deterministic. Additionally, *E. coli* test data Assembly_bp and Ncontigs statistics were identical across computing environments. The other discrepancy was caused by insufficient memory being available to Skesa^24^ on the Desktop computer causing two libraries to not assemble resulting in missing Skesa plasmid annotations (rows 33 and 35, columns 21 and 22 of GEA_ecoli_test_Ceres_metadata.txt in Supplementary Data S1). Importantly, these assembly failures were documented by the GEA pipeline log, which alerted the Desktop user. Apart from these two discrepancy sources (shovill final contig naming and Skesa memory requirements not being met), we expect that GEA will provide reproducible results for a given version of the pipeline and databases.

GEA has clear advantages and limitations relative to tools with similar goals. First, long available tools, such as nullarbor (https://github.com/tseemann/nullarbor) and TORMES^7^ have the distinction of an active user base, citations, and more time under development. Bacannot^25^ is a newer tool, which is container based like GEA. Software containerization increases reproducibility over OS specific source-compile-install-run and cross-platform package manager methodologies^17^. RSYD-BASIC^26^ is also a newer tool which produces a tabular output somewhat like that produced by GEA. The lack of a web server option is a limitation of GEA. However, having a batch command-line implementation of CGE tools was a central functionality driving the development of GEA. GEA also lacks phylogenetic methods, except to the extent that typing predictions are phylogenetically informative. Currently, GEA is only indicated for *E. coli* and *S. enterica*. The strongest advantages of GEA are a single dependency (Apptainer^27^), batch processing in an HPC environment proven by hundreds of successful analyses of illumina raw read libraries^21^, and the successful demonstration at the kilo-scale of a processing rate of ~ 18-s-per-*S.*-*enterica*-assembly as demonstrated in this report.

Shovill and Skesa can both handle low levels of contamination. The pipeline has been tested with a diversity of libraries from three different Illumina sequencing platforms at the USMARC Core Lab, but exhaustive testing on the types and degrees of contamination has not been conducted. Regardless, preprocessing or quality control of libraries should be unnecessary.

The Gammaproteobacteria name derivation of the GEA pipeline implies a much broader set of pathogens than are currently included. One noticeable impact of incorporating new species is a substantially smaller set of annotations for the added species, at least initially. This is because all other species have fewer applicable CGE tools relative to *E. coli*. This is surmountable in instances where there is a non-CGE tool available for the task (e.g. GEA uses SeqSero2^28^ for serotyping Salmonella since the CGE tool, SerotypeFinder^14^, does not serotype Salmonella). Other enhancements, which could be incorporated into GEA in the future include long-read sequence inputs, identification of additional genotypes/phenotypes as new CGE tools are released, and downsampling reads to accelerate Skesa assembly as is done with shovill. GEA is now available for other users with institutional HPCs for rapid characterization of large batches of *E. coli* and *Salmonella* genomes from diverse sample sources. GEA is available for download from github.com/Phylloxera/GEA-dev.

## Methods

### Pipeline

**Figure 1:**
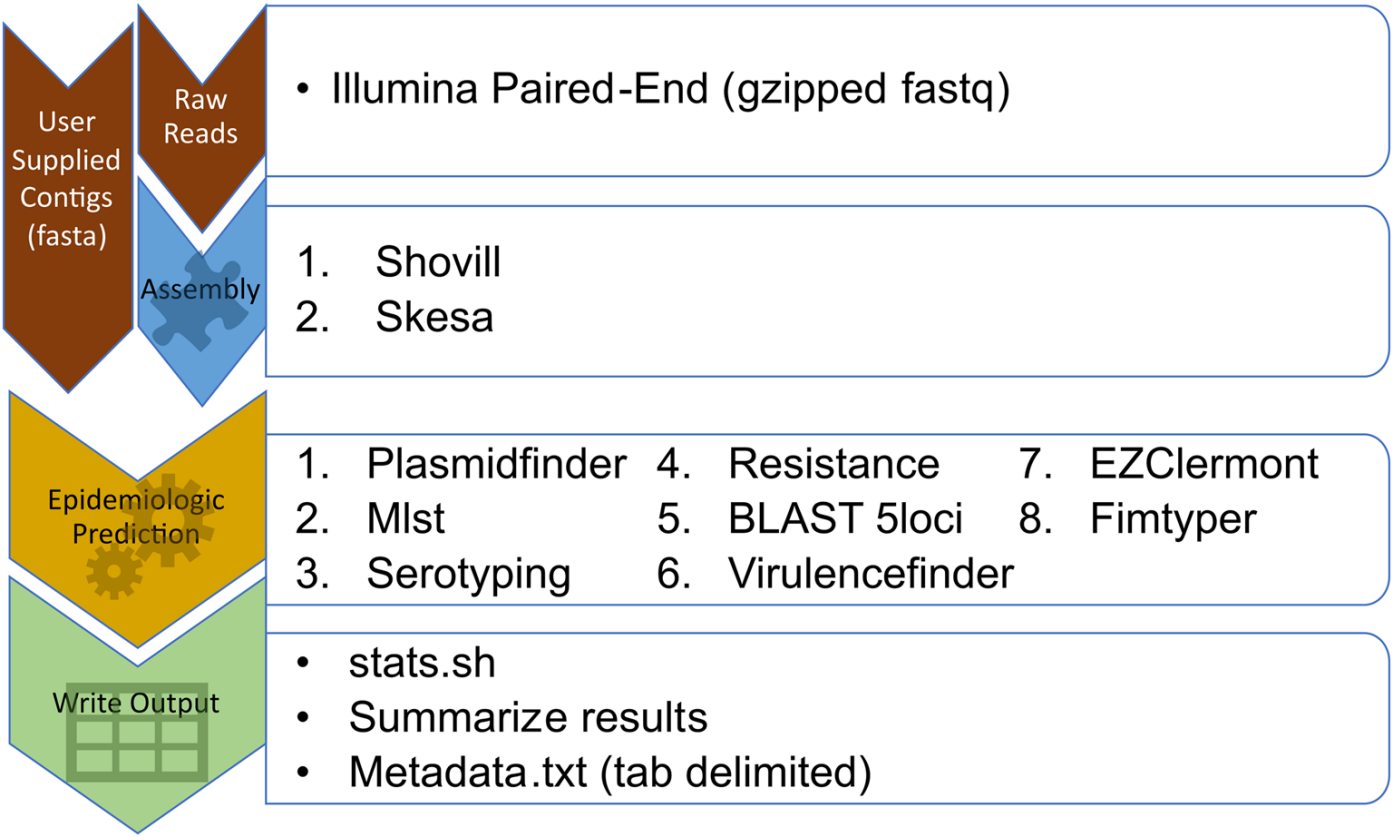
Sequential workflow carried out by the GEA pipeline.

The GEA pipeline implements the following steps in sequential order (Fig. 1). GEA processes the user command-line options and inputs. If the inputs are raw reads, the workflow proceeds to Assembly.

1. Assembly is first carried out by Shovill(https://github.com/tseemann/shovill) followed by SKESA^24^. Skesa is used for identifying complete plasmids and can circularize some novel small plasmids not yet on the plasmidfinder database.
2. Epidemiologic Prediction is conducted on shovill assemblies or user supplied fasta contigs. Local copies of the CGE tool and 5 loci databases (https://github.com/Phylloxera/5loci) are updated by the pipeline unless otherwise specified by the user. The databases are stored locally in the user’s home directory (e.g. $HOME/share/resfinder). BLAST^29^ is used as the search method for the CGE tools and to query the custom 5 loci databases.
  - PlasmidFinder^12^ (CGE)
  - MLST^11^ (CGE) is run. If the user does not specify the taxon, mlst is run against both databases and the species is determined by GEA.
  - Serotyping is conducted using SerotypeFinder^14^ (CGE—*E. coli*) or SeqSero2^28^ (*Salmonella*).
  - Resfinder4^13^ (CGE) is run against resfinder_db and pointfinder_db databases.
  - The 5 loci databases are queried with Blast.
  - VirulenceFinder^15^ (CGE—*E. coli*)
  - Ezclermont^30^ (*E. coli*)
  - FimTyper^10^ (CGE—*E. coli*)
3. The results are compiled and written to the tab delimited output file, metadata.txt. GC and N50 summary statistics are calculated by stats.sh(https://jgi.doe.gov/data-and-tools/software-tools/bbtools/bb-tools-user-guide/statistics-guide/) during results compilation and additional summary statistics are extracted from the shovill logs.

GEA is written in Bash. The current version has several new features relative to prior development iterations used in previous work.

1. The user can specify the taxon.
2. The user can specify whether to update their local copy of the databases.
3. The input data can be raw gzipped paired reads (fastq) or genome assemblies (fasta).
4. The following new tools have been added: Ezclermont, FimTyper, VirulenceFinder, and the 5 loci databases.
5. The container recipe utilizes SCIF^18^ for software environment modularity inside the software container.
6. The container, and pipeline are made available via https://github.com/Phylloxera/GEA-dev.

The shovill pipeline utilizes SPAdes^31^, Velvet^32^, Lighter^33^, FLASh^34^, SAMtools^35^, BWA-MEM^36^, KMC^37^, seqtk(https://github.com/lh3/seqtk), pigz(https://zlib.net/pigz/), Pilon^23^, Trimmomatic^38^ and samclip(https://github.com/tseemann/samclip). The container with the needed software was created using Apptainer^27^.

### Testing

GEA was tested on 3 linux high performance computers and a desktop computer with Hyper-V enabled on Windows 10 Professional (Table 1). The data used in testing were a single plate of 96 illumina *E. coli* raw read libraries from a long-term evolutionary study. Testing utilized the -u F option to query identical versions of the CGE databases and evaluate reproducibility across the compute environments.

### Demonstration

To demonstrate the pipeline at kilo-scale, GEA was run on 14,310 *Salmonella enterica* genome assemblies released during October, 2023. The assemblies were downloaded on November 6, 2023 using datasets(https://www.ncbi.nlm.nih.gov/datasets) with download genome options: taxon 28901,–include genome,–exclude-atypical,–released-after 10/1/2023,–released-before 10/31/2023,–assembly-source GenBank, and–dehydrated. Fasta files were moved to a single folder to be used as input. GEA was run on the Atlas high performance computer system of Mississippi State University and the US Department of Agriculture^22^ on November 11, 2023, with options: -t senterica, -u F, -r 336:00:00, -m 360G, and -c 48. Initial tests predicted a run time of 2–5 days.

### Supplementary Information

Supplementary Information.

## Data Availability

Underlying data: The test dataset used during the current study can be made available from the corresponding author on reasonable request. The demonstration dataset used during the current study are publicly available from the National Center for Biotechnology Information. The assemblies can be downloaded using the publicly available software, datasets (https://www.ncbi.nlm.nih.gov/datasets), with download genome options: taxon 28901,–include genome,–exclude-atypical,–released-after 10/1/2023,–released-before 10/31/2023,–assembly-source GenBank, and–dehydrated. Extended data: USDA-NAL: Supplementary Data S1. This project contains the following extended data:–GEA_ecoli_test_Ceres_metadata.txt. GEA Pipeline summary output (metadata.txt) for 96 *E. coli* Illumina libraries used as test data.–GEA_senterica_demo_Atlas_metadata.txt GEA Pipeline summary output (metadata.txt) for 14,310 *S. enterica* assemblies used as demonstration data. Extended data are available under the terms of the Creative Commons Attribution 4.0 International license (CC-BY 4.0).

## Code availability

The full pipeline (as an apptainer container) and files required for building the pipeline from scratch are available from: [https://github.com/Phylloxera/GEA-dev]. The version used in all analyses herein is 0.0.1-alpha.1. GEA License: [https://github.com/Phylloxera/GEA-dev/blob/main/LICENSE]. The 5 loci BLAST databases are available from: [https://github.com/Phylloxera/5loci]. 5 loci License: [https://github.com/Phylloxera/5loci/blob/main/LICENSE].
